# Contribution of prolonged-release melatonin and anti-benzodiazepine campaigns to the reduction of benzodiazepine and z-drugs consumption in nine European countries

**DOI:** 10.1007/s00228-012-1424-1

**Published:** 2012-11-01

**Authors:** Emilie Clay, Bruno Falissard, Nicholas Moore, Mondher Toumi

**Affiliations:** 1 Laboratoire de santé publique évaluation des systèmes de soins et santé perçue, University of the Mediterranean, Marseille, France; 2 INSERM, U-669 PSIGIAM, Paris, France; 3 INSERM U657, Service de Pharmacologie, Université Victor Segalen, Bordeaux, France; 4 Department of Decision, Sciences and Health Policy, University Claude Bernard Lyon I, Villeurbanne, France

**Keywords:** Insomnia, Benzodiazepines (BZD), Benzodiazepine receptor agonists, Z-drugs, Prolonged-release (PR) melatonin, Addiction

## Abstract

**Background:**

Benzodiazepines (BZD) and benzodiazepine receptor agonists (zolpidem, zaleplon, zopiclone, altogether Z-drugs) are most commonly prescribed for the treatment of insomnia. However, long-term use of BZD/Z-drugs is associated with major adverse events including, but not limited to, falls and fractures, domestic and traffic accidents, confusion, cognitive impairment, Alzheimer's disease and cancer. The prolonged use of these drugs is thought to be related to severe withdrawal symptoms and potential dependency. The chronic and extensive use of BZD/Z drugs has become a public health issue and has led to multiple campaigns to reduce both prescription and consumption of BZD/Z-drugs. Prolonged-release (PR) melatonin is the first of a new class of melatonin receptor agonist drugs that has demonstrated clinically relevant efficacy on improving quality of sleep and morning alertness, with a good safety profile.

**Objective:**

This study aimed to analyze and evaluate the impact of anti-BZD/Z-drugs campaigns and the availability of alternative pharmacotherapy (PR-melatonin) on the consumption of BZD and Z-drugs in several European countries.

**Methods:**

Annual sales data from nine European countries were extracted from the IMS sales database and analyzed to determine whether trends in use of these treatment options were attributed to campaigns and/or availability and affordability of safer alternatives on the market.

**Results:**

Campaigns aiming to reduce the use of BZD/Z-drugs failed when they were not associated with the availability and market uptake of PR-melatonin. The reimbursement of PR-melatonin supports better penetration rates and a higher reduction in sales for BZD/Z-drugs.

## Introduction

Insomnia is a disorder characterized by difficulties in initiating and/or maintaining sleep, nighttime or early awakenings, and nonrestorative or poor quality sleep for at least 1 month [1, 2]. Its diagnosis is further subdivided into primary insomnia with an absence of comorbid conditions, and secondary insomnia if it is associated with other conditions (physical, mental, environmental causes). Insomnia is associated with clinically significant daytime distress resulting in a reduced quality of life. Mental health problems, such as a reduction of cognitive abilities, memory and attention, as well as cardiovascular, respiratory and metabolic disorders are associated with insomnia [3]. Direct and indirect costs of insomnia represent a substantial societal economic burden [4]. The prevalence of primary insomnia ranges from 1 % to 10 % in the general population and up to 25–30 % in the elderly, [5–9] for whom treatment of insomnia is a clear medical need.

Benzodiazepines (BZD) and benzodiazepine receptor agonists (zolpidem, zaleplon, zopiclone, altogether Z-drugs) are most commonly prescribed for the treatment of insomnia [10, 11]. A meta-analysis of the risks and benefits of these therapeutic options in elderly patients reported statistically significant improvements in sleep, but also reported a statistically significant risk of adverse events [12, 13], including life-threatening ones [14]. Indeed these drugs are only approved by regulatory authorities for 2–4 weeks because of safety concerns. The Z-drugs, which unlike BZD are used exclusively for the treatment of insomnia, were thought to have a lesser tendency to induce physical dependence and addiction than BZDs [15], and are therefore widely prescribed for the treatment of insomnia, particularly in elderly patients [16–18]. Nevertheless, safety issues are still a matter of concern [19–25]. Long-term BZD and Z-drug use is not recommended, as tolerance and addiction can occur [26]. A population-based survey of patients using Z-drugs and BZD hypnotics found that Z-drug users were more likely to report that they had tried to stop using their hypnotic drug and were more likely to want to stop taking Z-drugs than BZD users. Adverse effects were reported in over 41 % of users with no difference between these two classes. Efficacy also did not differ between Z-drugs and BZD users [13].

In patients over 60 years of age, chronic BZD or Z-drug use carries the risk of exacerbations of pre-existing psychomotor or cognitive impairment, which may result in an increased risk of falls, motor vehicle collisions, household accidents or confusion and memory problems [27]. Recent studies have also pinpointed the potential increased risk of Alzheimer’s disease [28], cancer, and mortality [29] after chronic consumption of hypnotic drugs.

These safety concerns relating to the treatment of insomnia with hypnotic drugs, as well as the possibility of dependence, are a significant public health issue.

It has also been demonstrated that in some countries such as France, BZD and Z-drugs are overused and prescribed for a much longer time than the indicated 4 weeks [26, 30]. As a result, more and more health authorities in Europe are initiating policies and recommendations in order to decrease the consumption of BZD and Z-drugs [30–35]. However, the anti-BZD and Z-drug campaigns initiated in most countries have been unsuccessful, and despite the guidelines and national recommendations, the use of BZD and especially Z-drugs has continued to increase.

Prolonged-release (PR) melatonin is the first of a new class of drugs known as “melatonin receptors agonists,” and is a non-sedative hypnotic which has demonstrated clinically relevant efficacy on quality of sleep and morning alertness, with a good safety profile [36–39]. No evidence of dependence, withdrawal effects, rebound insomnia or negative influence on daytime alertness has been observed with its use [40, 41]. Several clinical trials demonstrated that PR-melatonin could help reduce BZD and Z-drugs consumption [42, 43].

PR-melatonin is only available as trade name Circadin, manufactured by Neurim Pharmaceuticals, Tel-Aviv, Israel. PR-melatonin 2 mg is the only alternative to BZD and Z-drugs, approved by the European Medicines Agency (EMA) in 2007 for patients aged 55 or over, as monotherapy for the short-term treatment (up to 13 weeks) of primary insomnia.

As PR-melatonin was launched in many European markets in 2008, it was interesting to evaluate how campaigns to decrease BZD and Z-drugs prescriptions affected consumption of these drugs in real life, with or without market uptake of PR-melatonin.

## Objectives

The objective of this study was to analyze and evaluate the impact of anti-BZD/Z-drug use campaigns and the availability of alternative pharmacotherapy (PR-melatonin) on the consumption of BZD and Z-drugs in several European countries.

## Methodology

The selection of European countries considered in the scope of this study was based on two criteria: countries having national or regional (in Spain) anti-BZD campaigns, and/or countries where PR-melatonin was launched and reached at least 4 % volume market share of the total insomnia market for N5B1 (NON-BARBITURATE PLAIN). The countries with anti-BZD campaigns were selected after reviewing their respective Ministry of Health, national public health, HTA agency, and regional health authorities’ websites. To determine countries with significant PR-melatonin market share, the IMS sales database was used. IMS Health is a global company that provides information, services and technology for the healthcare industry. The market volume is defined as the ratio of the number of PR-melatonin standard units sold to the total number of standard units sold for the treatment of insomnia. The Standard Unit (SU) is the smallest drug dose available on the market. The level of 4 % was considered to be a significant penetration rate—when the other hypnotics are generics with a price that is around 8 times lower, and the indication for PR-melatonin is only for insomnia patients aged over 55 while for the competitors are for all ages, 4 % in volume can reasonably be considered a significant penetration rate. It corresponds to approximately 20 % in value.

The European countries in the scope of this study were: Finland, Norway, Denmark, Sweden, Greece, France, the Netherlands, Spain and the United Kingdom.

This study was completed using the annual sale volumes of BZD/Z-drugs and PR-melatonin for each country in the scope, extracted from the IMS sales database. Data is expressed in SU. For each country, we studied the evolution of BZD/Z-drug sales volumes (together and separately) 3 years prior to the launch of PR-melatonin (at the end of 2007) and then 4 years after the launch of PR-melatonin (2011), as well as the evolution of PR-melatonin sales volumes. Additional parameters considered in the interpretation of the data were: the launch strategy of PR-melatonin (actively promoted/not promoted), product positioning and key messages, national or regional anti-BZD/Z-drugs campaigns (the type of campaign, their target and the recommendations), the penetration rate of PR-melatonin in 2011 and its reimbursement status compared to BZD/Z-drugs. As only the volume of sales is available in these databases, the assumption was made that the volume sold was equal to the prescribed and consumed volumes.

## Results

Table 1 presents market status of BZD/Z hypnotics and PR-melatonin for the countries within the scope of the study. The market trends for each country are depicted in Figs. 1, 2, 3, 4, 5, 6, 7, 8, 9 and are detailed below, by country.

**Table 1:** Market status of BZD/Z hypnotics and PR-melatonin for each country in the scope

Anti-BZD campaigns					PR-melatonin			BZD/Z-drugs		
Countries	Yes/No	year	Launch date	Promotion	Reimbursement	Volume market share in 2011 (SU%)	Price (€/tab)	Reimbursement	Volume market size in 2005 (million SU)	Zolpidem price (€/tab)
Finland	Yes	2005	January 2008	Actively promoted	No reimbursement	5.10 %	0.55	Partial reimbursement	105,962	0.07
Norway	Yes	2005	January 2008	Actively promoted	No reimbursement	4.50 %	0.53	No reimbursement	75,711	0.10
Denmark	Yes	2008	October 2007	Actively promoted	No reimbursement	3.70 %	0.55	No reimbursement	71,688	0.63
Sweden	Yes	2001	2008	Actively promoted	No reimbursement	1 %	0.62	Reimbursed	195,048	0.05
Greece	No	–	2008	Actively promoted	Reimbursed (automatic, at 75 %)	5.50 %	0.53	Reimbursed (automatic, at 75 %)	50,836	0.09
France	Yes	2008	June 2008	Not promoted	No reimbursement	<1 %	0.76	Reimbursed	766,207	0.16
Netherlands	Yes	2009	2009	Initially promoted	No reimbursement	<1 %	0.57	No reimbursement (since Jan 2009)	148,042	Not found
UK	Yes	2004	2008	Initially promoted	Reimbursed (automatic) not recommended by NICE^a^	<1 %	0.53	Reimbursed (automatic)	474,775	0.08
								Equal recommendation BZD and Z drugs by NICE		
Spain	Regional small campaigns	–	Not launched	NA	NA	NA	NA	Reimbursed	390,005	0.06

^a^In the UK, drug prices are freely chosen by pharmaceutical firms and reimbursed at 100 %. The control of practices is made through the recommendations of the National Health Service (NHS) after advice from the National Institute for Health and Clinical Excellence (NICE). In a general way, a product negatively recommended is not prescribed.

**Fig. 1:**
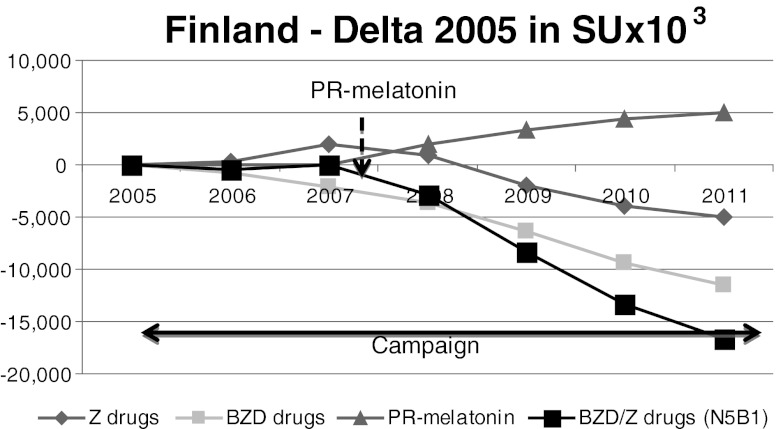
Finland. Despite the anti-BZD campaign, BZD/Z-drugs consumption remained stable until the introduction of PR-melatonin. At PR-melatonin’s launch in 2008, BZD and Z-drugs sales decreased substantially between 2008 and 2011

**Fig. 2:**
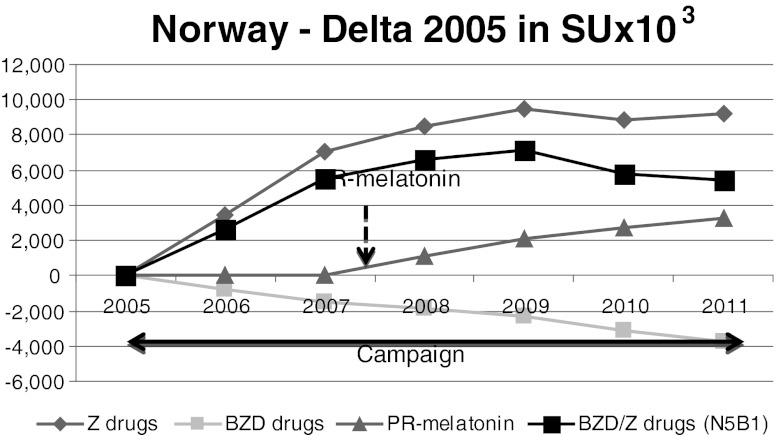
Norway. Despite the anti-BZD campaign prior to the launch of PR-melatonin, BZD/Z-drug net consumption increased between 2005 and 2007. BZD volume sales decreased whereas Z-drug sales increased. After PR-melatonin launch, BZD consumption decreased by 13.4 % between 2008 and 2011, whereas Z-drugs sales have stabilized. PR-melatonin reached a volume market share of 4.5 % in 2011

**Fig. 3:**
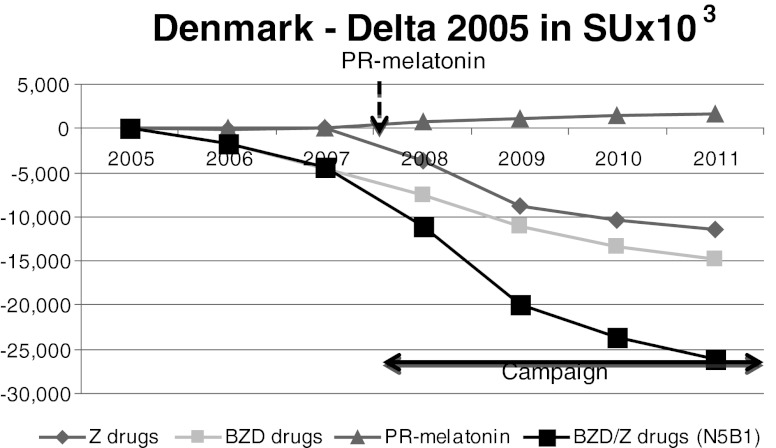
Denmark. Despite the absence of an anti-BZD campaign between 2005 and 2007, BZD sales started to decrease slightly whereas Z-drug sales remained at the same level. From 2008, when both PR-melatonin and a BZD campaign was launched, the BZD/Z-drugs sales decreased quite substantially

**Fig. 4:**
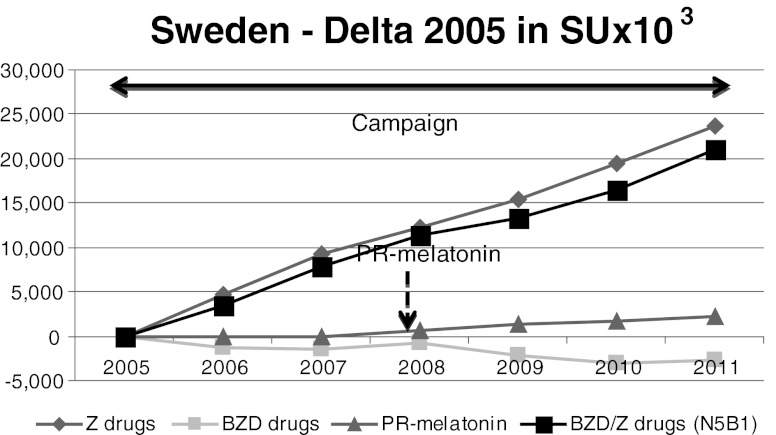
Sweden. The BZD consumption rate is quite stable whereas Z-drugs sales rose steadily from 2005 to 2011 despite the anti-BZD campaign. PR-melatonin sales were negligible

**Fig. 5:**
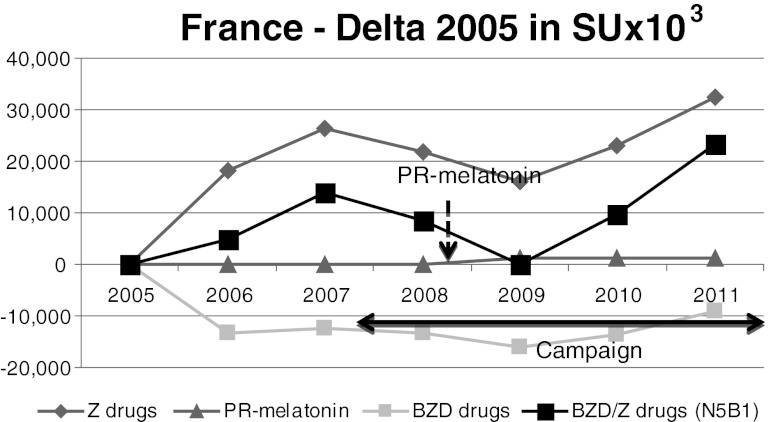
France. BZD sales decreased whereas Z-drug sales increased during the anti-BZD campaign. From 2007, both BZD and Z-drug sales remained stable despite the various reports and campaigns. PR-melatonin sales were negligible

**Fig. 6:**
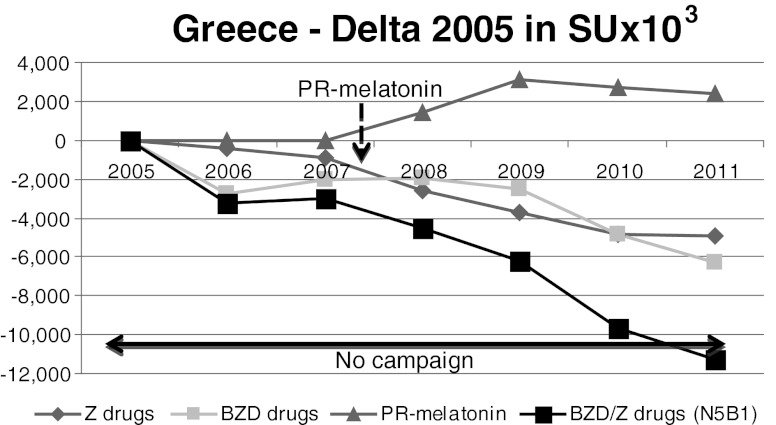
Greece. Between 2005 and 2007, there was a decrease of BZD/Z-drug consumption. After the PR-melatonin launch in 2008, this phenomenon accelerated, with a bigger decrease of the BZD/Z consumption between 2008 and 2011. The PR-melatonin’s penetration was progressive, finally reaching 5.5 % of volume market share

**Fig. 7:**
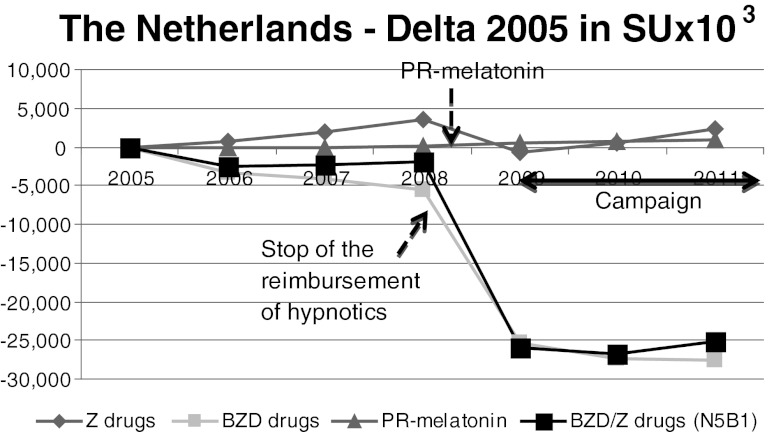
The Netherlands. The sales of PR-melatonin were negligible. Looking separately at BZDs and Z-drugs, BZD sales decreased sharply whereas Z-drug sales were stable between 2008 and 2011

**Fig. 8:**
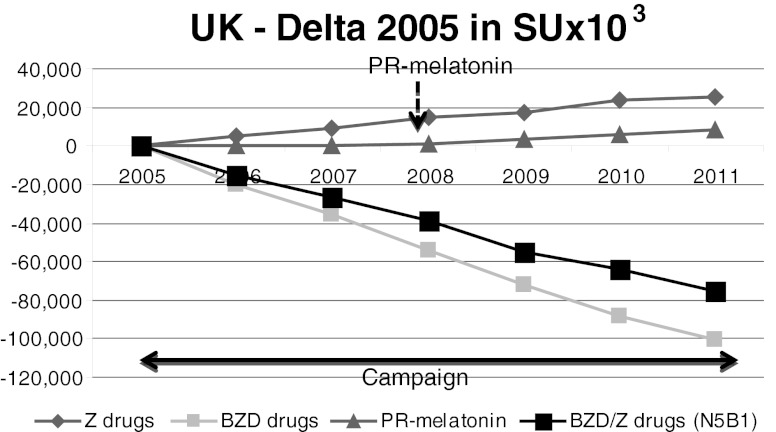
The UK. The decrease in BZD SUs between 2005 and 2010 was steady. Similarly, the sales of Z-drugs increased during this period. PR-melatonin sales were negligible in the UK

**Fig. 9:**
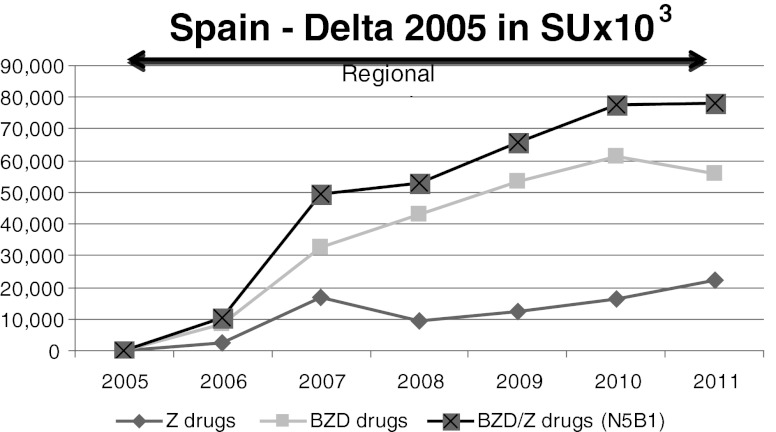
Spain. There was a substantial increase in BZD and Z-drug consumption between 2005 and 2011 and PR-melatonin was not on the market

### Finland

In Finland, health authorities have been carrying out an anti-BZD campaign since 2005 [33]. This government-driven campaign was supported by publications and guidelines with negative recommendations on BZD and Z-drug use. PR-melatonin was launched in January 2008 and marketed actively, but was not reimbursed, while BZDs and Z-drugs are partially reimbursed. The price of PR-melatonin in Finland is about eight times higher (0.55€ ex-factory/day) than the mean price of BZD/Z-drugs (0.07€ ex-factory/day). Nevertheless, patients are used to paying for medications and the impact of price on patient decisions is limited.

As depicted in Fig. 1, BZD/Z-drug consumption remained stable between 2005 and 2007. More precisely, BZD sales decreased in proportion to the increase in Z-drug sales. Since the launch of PR-melatonin in 2008, BZD and Z-drugs sales have decreased substantially, with a reduction of up to 20.2 % between 2008 and 2011. PR-melatonin sales increased gradually between 2008 and 2011, to reach 5.1 % in volume (SU) market share and 27.1 % in value market share in 2011. In 2011, 5 million SUs of PR-melatonin were sold whereas the annual sales of BZD/Z-drugs reduced by 16.6 million SUs compared to 2005.

### Norway

In Norway, an important anti-BZD government driven campaign was initiated in 2005 [44]. This campaign was supported by guidelines and especially focused on the issue of driving and BZD/Z-drug consumption [44]. PR-melatonin was launched in Norway in January 2008, but was not reimbursed. BZD and Z-drugs were not reimbursed as well.

Despite the anti-BZD campaign prior to the launch of PR-melatonin, BZD/Z-drug net consumption increased by 7.3 % between 2005 and 2007. BZD volume sales decreased (9.3 %) whereas Z-drug sales increased (11.7 %) during this period. After its launch in January 2008, PR-melatonin reached a volume market share of 4.5 % and a value market share of 21 % in 2011. BZD consumption decreased by 13.4 % between 2008 and 2011, whereas Z-drugs sales stabilized (+1.0 %).

### Denmark

The Danish Institute of Rational Pharmacotherapy (within the Danish Medicines Agency) started a campaign against the use of BZD and Z-drugs in 2008, with three brochures provided to physicians, citizens and pharmacies [45]. They pinpoint the fact that long-term consumption of BZD and Z-drugs is associated with health risks. Approximately 100,000 people in Denmark permanently consume BZDs and are addicted to them [45].

PR-melatonin was launched in October 2007, without reimbursement status, similar to BZD/Z-drugs that are also not reimbursed. PR-melatonin was not recommended as first-line therapy in the treatment of primary insomnia [46]. Since Denmark citizens are used to paying for insomnia drugs, the non-reimbursement status did not differentiate PR-melatonin from BZD/Z-drugs.

Between 2005 and 2007, Z-drug sales remained at the same level whereas BZD sales started to decrease slightly, despite the absence of an anti-BZD campaign. In 2008, a campaign was launched alongside the introduction of PR-melatonin. PR-melatonin’s market share reached 3.7 % of volume in 2011 and 21 % in value market share in 2011. From 2008, the sale of BZD/Z-drugs decreased quite substantially (by 24.7 %).

### Sweden

In Sweden, all BZD and Z-drugs are reimbursed. An anti-BZD campaign was launched in 2001 [47], resulting in a stagnation of the sales of BZD, with an increase of Z-drugs sales. PR-melatonin was launched but was the only non-reimbursed hypnotic. It represented only 1 % of volume market share and 11 % of value market share in 2011, as shown in Fig. 4. The BZD consumption rate was quite stable (−3.2 %) whereas sales of Z-drugs rose steadily (+20.5 %) from 2005 to 2011.

### France

In France, health authorities are concerned about the overuse of BZD and Z-drugs. An initial report warning about the use of BZD/Z-drugs was issued in the early 90s [48], followed by a series of reports issued upthrough 2010 and many campaigns, the most prominent being in 2008 [26]. The risks of these products are well known, and the Haute Autorité de Santé (HAS) is trying to reduce their consumption. The HAS has published detailed recommendations on how to help patients withdraw from the use of BZD: “Psycho SA - Plaintes du sommeil-Insomnie 2010” [26]. Also, the Agence Française de Sécurité Sanitaire Produits de Santé (AFSSaPS) addressed the high levels of BZD consumption in France through a review of the last 10 years [30], and tried to reduce consumption by controlling use and strengthening the measures already initiated to promote the appropriate consumption of BZD and Z-drugs.

PR-melatonin was introduced to the French market in June 2008. The product was not actively promoted in France, since it was not reimbursed while all other hypnotics were reimbursed. The price was eight times higher than the mean BZD price. As French patients are not used to paying for their medication “out of pocket,” there was a substantial disincentive for choosing PR-melatonin prescriptions.

As observed in Fig. 5, the sales of BZD/Z-drugs did not change significantly following the recommendations of the HAS, with the global variation of +1.8 %. More precisely, BZD sales decreased by −6.0 % whereas Z-drug sales increased by +4.7 % during the campaign. From 2007, both BZD and Z-drug sales remained stable despite the various reports and campaigns issued by the health authorities. PR-melatonin sales were negligible.

### Greece

No anti-BZD campaign has been initiated in Greece. PR-melatonin was launched in January 2008 and reimbursed like all other hypnotics.

Before PR-melatonin’s launch, consumption of BZD/Z-drugs decreased by 5.9 % between 2005 and 2007 (Fig. 6). After the launch, this phenomenon accelerated, with a decrease of 14.5 % in BZD/Z-drug consumption between 2008 and 2011. PR-melatonin penetration was progressive, reaching 5.5 % of volume market share and 28 % of value market share in 2011. Compared to sales in 2005, the annual sales of BZD/Z-drugs decreased by more than 11 million SUs. Approximately 2.4 million SUs of PR-melatonin were sold during 2011.

### The Netherlands

The Netherlands College of General Practitioners recommended that BZD and Z-drugs should be prescribed only for short courses, and should generally be avoided in elderly patients. Indeed, around 30 % of Dutch patients using these compounds are chronic users [49]. In January 2009, the reimbursement status of BZD and Z-drugs changed, and became excluded from the Dutch reimbursement schemes. The aim of this change was to reduce the use of these medications for chronic use, and to limit the health care expenditures (high level of costs due to the volume of BZD use). After ending reimbursement, the Dutch Foundation for Pharmaceutical Statistics reported a 16 % reduction in the overall use of BZDs and Z-drugs [50].

In the Netherlands, PR-melatonin was launched without a reimbursement status. As shown in Fig. 7, the sales of PR-melatonin were negligible. Looking separately at BZDs and Z-drugs (Fig. 7), BZD sales decreased by −19.4 % whereas Z-drug sales were stable (−3.6 %) between 2008 and 2011 and since the ending of reimbursement in 2009, both BZD and Z-drugs sales are stable.

### United Kingdom

In the United Kingdom, recommendations to restrict BZD and Z-drug usage were published on 2004, by the Department of Health (DoH) [51]. In this recommendation, doctors are warned that benzodiazepines should only be prescribed for short-term treatment, in light of evidence of problems associated with long-term use.

PR-melatonin was launched in 2008 and automatically reimbursed in the UK, but was not recommended by the National Institute of Clinical Excellence (NICE) and the Scottish Medicines Consortium (SMC).

The decrease in BZD SUs between 2005 and 2010 was steady, up to 31.7 % less, as shown in Fig. 8. Concurrently, the sales of Z-drugs increased during this period (+7.4 %). PR-melatonin sales were negligible in UK.

### Spain

Campaigns were conducted at regional levels; in addition, PR-melatonin was approved, but not reimbursed and thus was not put on the market.

As shown in Fig. 9, there was a substantial 20 % increase in BZD and Z-drug consumption between 2005 and 2011.

## Discussion

The results of this analysis suggest there are three common groups among the studied countries, with different BZD/Z-drug consumption trends:

- Countries where the sales of BZD and Z-drugs decreased since 2007: Greece, Finland and Denmark.
  In Greece there was no anti-BZD campaign before the launch of PR-melatonin, and the consumption of the BZD and Z-drugs was stable. BZD and Z-drug consumption decreased by 14.5 % over 3 years after the introduction of PR-melatonin in the market. The decrease in BZD/Z drug consumption since 2008 can thus be attributed to the launch of PR-melatonin and its considerable market penetration. On average, an increase in 1 SU of PR-melatonin was associated with a decrease of about 4 SUs of BZD/Z-drugs.
  The combined launch of PR-melatonin and anti-BZD campaigns in Finland and Denmark seems to be associated with a reduction of BZD/Z-drugs usage. This decrease is concomitant with the penetration of PR-melatonin on the market and the campaign implementation. Again, uptake of 1 SU PR-melatonin in Finland was associated with a decrease of 3 SUs of BZD/Z drugs consumption in this country.
- Countries where the sales of BZD decrease while Z-drugs increase: Norway, the Netherlands and the UK. In these countries the anti-BZD campaigns seem effective for BZDs, but essentially resulted in a shift in prescription patterns towards Z-drugs.

In Norway, there was an overall increase in BZD/Z drugs consumption since 2005 but the BZD sales decreased in favor of Z-drugs. Since PR-melatonin was launched, the increase in Z-drug sales stopped and the consumption was stabilized, as if the switch from BZDs gradually shifted from Z-drugs to PR-melatonin.

The same evolution of BZD and Z-drug sales was observed in the Netherlands, but the decrease in BZD sales was mostly related to the change in the reimbursement status, suggesting that BZD/Z drug consumption in this country is price sensitive and reimbursement itself has some encouraging effect on hypnotic drug consumption. Nevertheless, Z-drug sales remained stable between 2009 and 2011. PR-melatonin sales did not rise considerably in the Netherlands perhaps because it is more expensive than the other drugs and is not actively promoted in this country.

In the UK, a decrease was seen only in BZD. There was a steady increase in Z-drug use of up to 7.3 % in 2011, although NICE has issued the following recommendation: "It is recommended that, because of the lack of compelling evidence to distinguish between zaleplon, zolpidem, zopiclone or the shorter-acting benzodiazepine hypnotics, the drug with the lowest purchase cost (taking into account daily required dose and product price per dose) should be prescribed” [52]. Possibly, higher market acceptance of PR-melatonin might gradually change this situation as seen in Norway.

- Countries where the sales of BZD were stable and Z-drug use increased, resulting in overall increases in BZD and Z-drug sales despite anti-BZD campaigns: France, Sweden and Spain.
- In these countries the anti-BZD/Z-drug campaigns that were sometimes quite intense and long lasting (like in France) had no or very limited impact on prescription levels. As BZDs and Z-drugs are reimbursed while PR-melatonin is not, and these markets are reimbursement-sensitive, PR-melatonin was not commercially launched in France and was not put on the market in Spain.

Although real-life outcomes are difficult to interpret, as many factors could contribute to the occurrence of the outcome, some conclusions may be drawn with a reasonable level of certainty in the light of this research.

In countries where BZD/Z-drug campaigns were launched and PR-melatonin was not promoted nor prescribed, all campaigns failed to reach the desired outcome. This was the case for France, Spain, and Sweden.

In Greece, where no campaign was initiated, the sales reduction of BZD/Z-drugs was concomitant with PR-melatonin uptake. The case of Greece is a robust argument in favor of the role of PR-melatonin in the reduction of BZD/Z-drug sales.

In Finland and Denmark, the concomitant launch of BZD/Z-drug campaigns and PR-melatonin made it difficult to weight the impact of each factor on the reduction of BZD/Z-drug sales. However, in Finland the 3-year campaign from 2005 to 2008 ended with no appreciable effect at the launch of PR-melatonin. The drop of BZD/Z-drug sales clearly followed the uptake of PR-melatonin.

It should be noted that a standard unit is the smallest available drug dose. For PR-melatonin, an SU is the defined daily dose (DDD), as there is only one dosage available on the market. For BZD/Z-drugs there are often several dosages and therefore more than one SU may account for a DDD. Thus, the volume of BZD/Z-drugs in SUs replaced by the sales of PR-melatonin is higher than the raw number of SUs of PR-melatonin sold. In addition, the lower SU volumes of sold PR-melatonin as compared to unsold BZD/Z-drug SU volumes may in part reflect the fact that PR-melatonin can be discontinued without difficulty while BZD/Z-drugs cause withdrawal, tolerance and dependency, making discontinuation very difficult and causing abuse.

In Norway, the prescription shift of BZD toward Z-drugs stopped suddenly when PR-melatonin was launched. PR-melatonin appears to be a successful alternative option to Z-drugs.

In the UK and the Netherlands, in the absence of PR-melatonin uptake the reduction of BZD sales was associated with an increase of Z-drug use. The objective of total reduction was not achieved. The shift cannot be considered a success of the anti BZD/Z drug campaign, as the risk associated with Z-drugs is not considered significantly different from BZD in most studies [27]. Some studies found Z-drugs to be even worse [53].

The marketing strategy toward positioning and promotion of pharmaceutical products is a critical element of the medical practice [54]. In this case in Greece, Finland, Denmark and later Norway, unlike Sweden, PR-melatonin was perceived as an option to help chronic users withdraw from BZD/Z-drugs. Although this was not the only positioning, it was an important element of the marketing strategy also leading to volume market shares of 4–5.5 %. In those countries, the sales of PR-melatonin were associated with a decrease of BZD/Z-drug sales.

The lack of success of anti-BZD/Z-drug campaigns in the absence of an alternative pharmacological treatment option (France, Sweden) raises the question of the utility of such campaigns. Even if BZD drugs were actually reduced in countries like the UK and Norway, they were always associated with a shift in prescription toward another pharmacological agent, namely Z-drugs alone (UK) or Z-drugs followed by PR-melatonin when it became available (Norway). When both PR-melatonin and Z-drugs were available the prescriptions were consistently channeled toward PR-melatonin, resulting in a net decrease of the whole sedative hypnotics class including BZDs and Z-drugs (Finland and Denmark). The availability on the market of pharmacological alternative options to replace BZD/Z-drugs appears to be a critical factor for success of such campaigns. In the Netherlands, despite the fact that reimbursement was ended for both BZD and Z-drugs, there was a shift toward Z-drug prescription for some of the patients. It is unclear how the other patients were managed. Additionally, no information is available on alternative pharmacological or nonpharmacological prescriptions. Therefore, it is not possible to appreciate potential harm associated with this shift in practice.

The adoption of PR-melatonin as a treatment option is also important for BZD/Z drug use in countries where the product is reimbursed (e.g. the UK and Greece). In the UK the PR-melatonin launch was not associated with an HTA recommendation and sales didn’t take off, as the recommendation is a strong driver of general physician (GP) prescriptions. However in Greece, where the adoption of PR-melatonin was high, so was the decrease of sales of BZD/Z-drugs despite the lack of campaigns.

In Spain, where PR-melatonin was not available and the campaign was mild and geographically limited to regions, and in France were the campaign was intense and national, the sales of BZD/Z-drugs still tended to increase.

The findings of this study are consistent with a small size, double-blind randomized clinical trial that has shown the role of PR-melatonin in helping patients to withdraw from BZD/Z-drug use [43]. It is also supported by an observational study showing a low rate of reinitiation of BZD/Z-drug after a course of PR-melatonin when patients were previously treated by BZD/Z-drugs [42].

The study has also some limitations.

No country without any campaigns and without a significant PR-melatonin existence was selected as control country. However, it is established that prescription habits of doctors are deeply anchored and without any intervention, no change occurs [55]. Moreover, in order to compare trends with and without campaigns, some countries can be their own control by comparing the period before with the period after the launch of the campaign, for example France.

We used a database that is solely based on sales data and not prescriptions. We assumed that sales figures are a good proxy of what is consumed even if it is clearly higher. Indeed, some sold drugs are not then consumed by the patients. However, we assumed that the proportion of drugs sold and actually consumed by patients is the same, whatever the product. There are no reasons to believe that the proportion is different between products.

Unlike Z-drugs, BZD could be used for other indications such as epilepsy or anxiety [56, 57]. However, we only considered N5B1 IMS classification in this study, which is for non-barbiturate drugs and is mostly used for insomnia, while BZDs used for other indications are more likely to be reported under other IMS classes, such as N5C (Antidepressants and Anxiolytics).

We didn’t collect and analyze whole promotional materials, but we relied on interviews of the company’s marketing leader that provided a clear picture of the positioning and promotion of PR-melatonin. The dichotomy of positioning (or not) of PR-melatonin to help patients discontinue BZD/Z-drugs was quite clear. Moreover, the people interviewed were not aware of the ultimate research goal and therefore were unlikely to be biased.

There were no prescriber interviews to appreciate the drivers of their prescriptions, and the role of campaign and PR-melatonin promotion. This could limit the interpretation of the reasons for prescribing PR-melatonin as a means of discontinuing BZD/Z-drug use. In this research we were not assessing the causal relationship but just the existence or not of a relationship.

Finally, we didn’t perform a thorough review of campaigns to appreciate the reasons for failure, as this wasn’t the objective of our research.

## Conclusion

Long-term prescription of BZD/Z-drugs is associated with major adverse events including, but not limited to, falls and fractures, domestic and traffic accidents, confusion, cognitive impairment, Alzheimer's disease and cancer. The prolonged use of these drugs is thought to be related to severe withdrawal symptoms and potential dependency. The chronic and extensive use of BZD/Z-drugs has become a public health issue and led to multiple campaigns to both reduce the prescription of BZD/Z-drugs and achieve discontinuation of long-term treatment. In our research, we observed the failure of those campaigns when they were not associated with the availability and uptake of sales of PR-melatonin. The reimbursement of PR-melatonin may support a better market penetration and a higher reduction of sales of BZD/Z-drugs. The non-reimbursement of BZD/Z-drugs appeared to have no effect on Z-drug prescription, and even showed an increase in prescription during 2011. When considering campaigns aiming to limit the usage of BZD/Z-drugs, policy makers should carefully consider the availability of reimbursed effective and safe pharmacological alternatives.
